# Accuracy of 3D facial scans: a comparison of three different scanning system in an in vivo study

**DOI:** 10.1186/s40510-023-00496-x

**Published:** 2023-12-25

**Authors:** Federica Pellitteri, Fabrizio Scisciola, Francesca Cremonini, Matilde Baciliero, Luca Lombardo

**Affiliations:** 1 Department of Orthodontics, University of Ferrara, Via Luigi Borsari, 46, 44121 Ferrara, Italy; 2 Postgraduate School of Orthodontics, University of Ferrara, Via Luigi Borsari 46, 44121 Ferrara, Italy

**Keywords:** Facial scanner, Digital orthodontics, 3D comparison, Facial scanner accuracy

## Abstract

**Background:**

The aim of the study was to compare the accuracy and reproducibility of three different 3D facial scanning systems, relying, respectively, on stereophotogrammetry, structured light and a smartphone app and camera.

**Methods:**

Thirty subjects have been scanned with three different facial scanning systems, stereophotogrammetry, structured light and a smartphone app and camera. Linear measurements were compared with direct anthropometries measured on the patient's face, while the study of areas (forehead, tip of the nose, chin, right and left cheek) was evaluated by overlapping scans using the Geomagic Control X program. Statistical analyses were conducted using IBM SPSS v28 software.

**Results:**

The ANOVA test was used to compare linear distances and direct anthropometry measurements, revealing statically significant values for all distances investigated, especially for the Face Hunter scanner, except for the Prn–Pog′ distance (*p* = 0.092). The three facial scans were superimposed pairwise almost the 100 per cent of the overlapping areas fell within the tolerance limits for all three comparisons analysed. The chin was the most accurately reproduced, with no differences among scanners, while the forehead proved to be the least accurately reproduced by all scanners.

**Conclusions:**

All three acquisition systems proved to be effective in capturing 3D images of the face, with the exception of the Face Hunter scanner, that produced statistically significant differences in linear measurements for the distances Tr–Na′ and Zyg–Zyg with respect to direct anthropometric measurements.

## Introduction

The methods for evaluating facial morphology and metrics have undergone a revolution in recent decades thanks to new 3D image-acquisition technology. As a result, cutting-edge techniques for 3D analysis have replaced conventional 2D photograph analysis, and enable the measurement of surface areas, volumes and angles, as well as the registration and superimposition of 3D surfaces [1–4]. The main benefit of 3D facial scans is their ability to capture the patient’s entire face in 3D without the need to expose the subject to radiation and without the angular errors that can occur with 2D methods. On facial scans, it is possible to quantify linear and angular distances, superimpose different areas, and study volume changes in specific areas of the face.

The literature increasingly reports that 3D facial scanners demonstrate a high degree of precision and accuracy, making them suitable for use in the field of dentistry [1, 3]. In particular, it allows the assessment of facial changes in growing children, analysis of facial characteristics in patients with pathologies, evaluation of asymmetries, and the study of soft tissue in orthognathic surgery patients. [1, 3] In fact, nowadays soft tissues are one of the most important factors in treatment planning and must be carefully analysed by the orthodontist. Correct diagnostic registration of soft tissues is therefore of utmost importance. [4, 5]

Advancements in technology, such as the advent of structured light systems or stereophotogrammetry, have made it possible for 3D imaging systems to be extremely less time-consuming and more accessible from a learning curve perspective. The structured light system allows the acquisition of the surface of the patient's face through continuous light emission, which undergoes distortions and deformations due to the irregularity of the scanned surface [5]. Stereophotogrammetry, on the other hand, consists of a multi-camera system that simultaneously captures two or more images of the same patient from different angles. This technique has undeniable advantages, including preventing involuntary facial or head movements and facial expressions from altering the accuracy of the scan. [1]

In addition, the development of 3D imaging systems for smartphones has allowed their use to extend to the medical and health fields. The quality and performance of the cameras within smartphones has improved to such an extent that they now have the technology to capture the subject's face in three dimensions. Among the advantages this technology offers clinicians is certainly that it is inexpensive, practical and accessible [5, 6].

Linear measurements on 3D facial scans have been widely studied in the literature. High accuracy has been reported for their linear measurements, with a mean error of between 0.2 and 1 mm. [7–11]In addition, Pellitteri [3] and Wang [12] analysed the reproducibility of 3D imaging of different areas of the face. They obtained satisfactory results for the middle and lower thirds of the face, with the highest average value (almost 60%) for the right and left cheeks, being highly reproducible. The figures for the tip of the nose and the chin were also good, but the forehead was associated with lower reproducibility values than other areas. [3]

Despite the literature on the accuracy of individual 3D imaging systems, there are many options on the market, and inter-device repeatability still needs to be verified, analysing scans obtained via different devices and comparing the relative performance of each.

Hence, this study was designed to compare the accuracy and reproducibility of three different 3D facial scanning systems, relying, respectively, on stereophotogrammetry, structured light and a smartphone app and camera, by comparison with direct anthropometry on the subjects’ faces and in overlays of 3D areas of the face. The first, the Vectra M3 3D Imaging System (Canfield Scientific, Parsippany, NJ.) is a static device that captures three shots simultaneously via three cameras. The system has a 3.5 ms acquisition time, and the stereophotogrammetry is guided by integrated intelligent and adaptable flash units and can deliver a 1.2 mm of geometrical resolution [1]. The second, the Face Hunter (Zirkonzahn, Gais [BZ], South Tyrol, Italy), is a structured light system whose scanner projects a light pattern onto the model, and analyses how the light deforms on the surface to map its geometry. Using a scan speed of 0.3 s, Basler ac780 and ac1600 cameras, and a Dell M318WL projector [3]. The third and final system compared, the Bellus 3D Dental Pro App (Bellus 3D, Inc, Campbell, Calif), specifically the iPad Pro and iPhone X versions of the Bellus 3D Dental Pro software, works on Apple devices with a TrueDepth camera running iOS 12.2 or later. [3]

## Materials and methods

After approval by the University of Ferrara institutional review board and informed consent release, 30 volunteers—postgraduate students at the University of Ferrara Department of Orthodontics—8 men and 22 women between the ages of 25 and 34 years, were recruited for the study. The inclusion criteria were non-growing patients, older than 25 years. Subjects with deformations, previous trauma to the facial area, facial plastic surgery or skin blemishes were excluded from the study. Men with beards were also excluded, due to the inability of stereophotogrammetric devices to acquire areas fully covered by hair.

Each participant was measured manually, and scanned using the three devices—the Face Hunter facial scanner, the Dental Pro facial scan application, and the Vectra M3 3D Imaging System—on the same day. Three calibrated operators performed all the scans and verified the correct processing of the 3D image. A few minutes elapsed between the scans made by the different devices.

On the day of the scans, each participant was instructed to take off any jewellery they might have been wearing, and their hair was pulled back through a band to reveal their forehead and ears.

Using a specific cross-shaped mould, six reference markers were applied to each subject's face at the cephalometric points, then to be scanned (Table 1). The distances between the Tr (trichion)–Na^′^ (soft tissue Nasion), Na^′^–Prn (Pronasion), Prn–Pog′ (soft tissue Pogonion) and left–right Zyg (Zygomatic) points were manually measured as a reference using a digital calliper. After two hours, the linear measurements were repeated by a different operator, in order to account for operator measurement errors. To prevent the body and head from moving backward or forward, and to preserve the head’s proper natural position, all participants were made to sit on a chair with a backrest [15–17]. In order to prevent variations in head position or facial expression that would distort the measures of the study, a thorough quality-control evaluation was carried out.

**Table 1:** Definition of the six cephalometric points

Cephalometric point	Definition
Trichion (Tr)	The most superior midline point on the forehead, located at the hairline where the forehead meets the scalp. It is anatomically situated at the junction of the frontal bone and the anterior hairline
Soft tissue Nasion (Na’)	The midpoint of the junction between the forehead and the nose. It corresponds to the most anterior point on the profile where the nasal dorsum transitions into the glabella, which is the smooth area between the eyebrows
Pronasion (Prn)	The most projected point of the nasal tip, which is commonly referred to as the apex of the nose
Left Zygion (L-Zyg)	The most lateral point on the contour of the left zygomatic arch. It corresponds to the outermost point of convexity of the left cheekbone area when viewed from the side. This point is typically located in line with the outer corner of the left eye
Right Zygion (R-Zyg)	The most lateral point on the contour of the right zygomatic arch. It corresponds to the outermost point of convexity of the right cheekbone area when viewed from the side. This point is typically located in line with the outer corner of the right eye
Soft tissue Pogonion (Pog’)	The most projected point of the chin

The Face Hunter scanner from Zirkonzahn was used to create the first scan. All individuals were required to maintain an arm’s length distance from the scanner while seated on a chair with a backrest. The patient was appropriately positioned in front of the scanner camera by the operator, who also ensured that the correct position of the subject was duplicated on the computer screen. In order to create a single 3D scan, a technician generated five static images of the face with occluded arches: one from the front, one from each side, and left and right ¾ profiles. The reference markers in each of these scans were then aligned by the technician and processed by the software.

The Dental Pro software from Bellus 3D was downloaded from the Apple App Store on an iPhone 12 Pro Max (Apple Inc.) for the second scan. Each participant was instructed to hold up the phone with their dominant hand, maintaining their arches in occlusion throughout the scan. The software automatically adjusted the exact tilt of the head, and the distance between the subject and the phone. It also showed correct positioning via a green oval around the subject’s face on the screen—a red oval indicated erroneous positioning. Once the face was centred, the subject was then instructed to turn their head by a robotic voice, until the imaging app had acquired a complete set of data.

For the final scan, by static Vectra M3, the stool was placed in front of the three-pod camera system. The scans were taken according to the specific conditions suggested by the manufacturer, 20–30 cm below the subject’s face. Specifically, two images were taken 45° to their right and left side, and one in the frontal position, just seconds from each other. The device was connected to a laptop throughout the whole acquisition session so that 3D reconstruction accuracy could be checked.

**Fig. 1:**
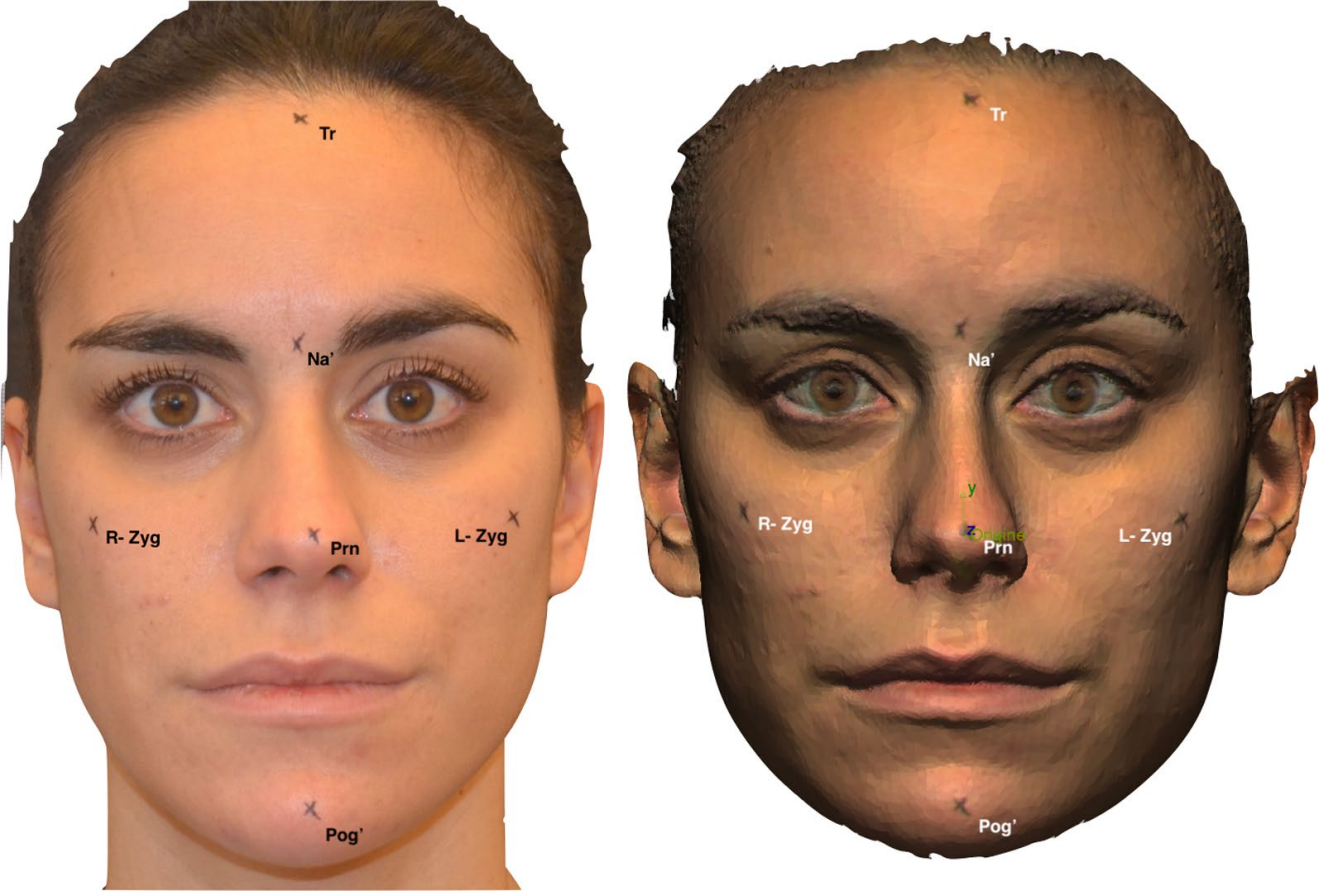
Frontal photograph and facial scan of the subject with reference points in order from top to bottom: Tr (midline of hairline), Na’ (point on soft tissue over nasion), Prn (soft tissue point on tip of nose), L–R Zyg (lateral point of zygomatic arches), Pog′ (soft tissue over pogonion)

All the 3D images taken already landmarked with the cephalometric points used for the direct measurements (Fig. 1).

**Fig. 2:**
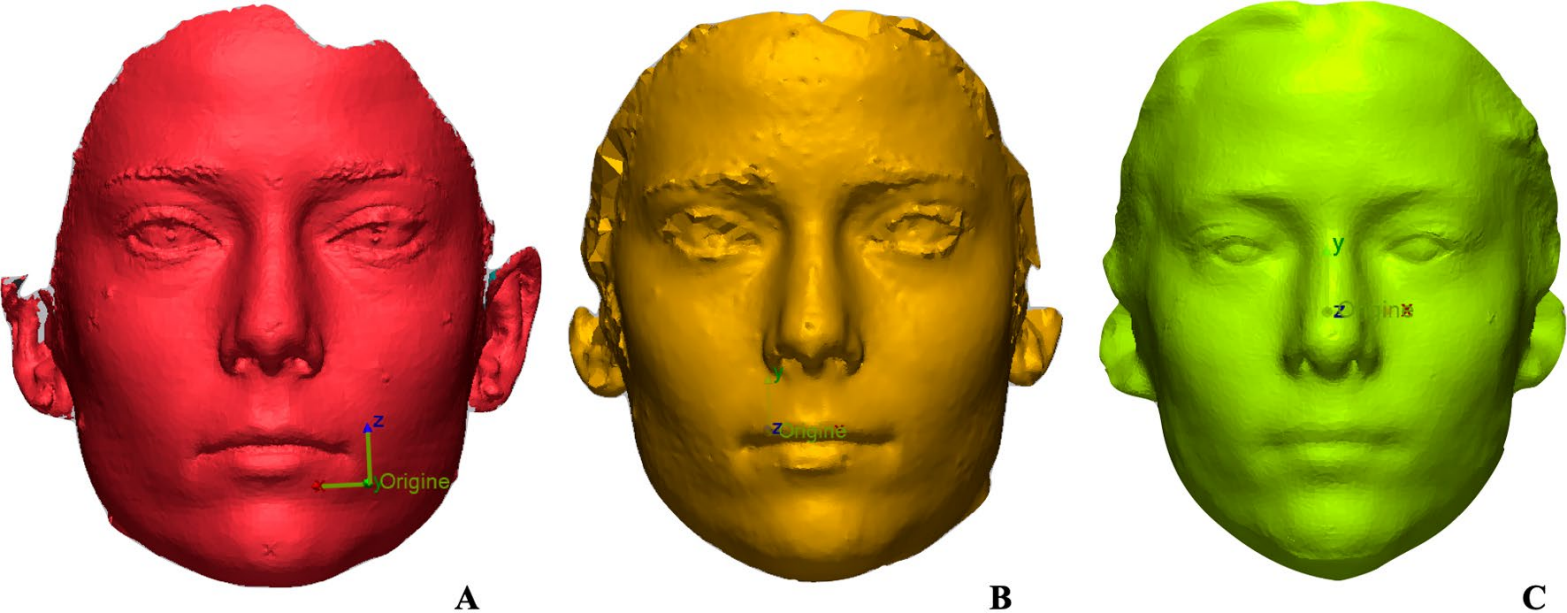
Facial scan of the same subject with Face Hunter (**A**), Vectra (**B**) and Bellus 3D (**C**)

Subsequently, the distances between the cephalometric reference points on each of the scans were calculated using digital measurement software, and sets of measurements were compared with one another and the manual reference. After 2 days, the linear measurements on the face scans were repeated by another operator to eliminate possible measurement errors and verify the repeatability of the measurements. Then, scans of the same subject were uploaded to Geomagic X Control software (3D Systems Inc, Rock Hill, SC) (Fig. 2) in order to verify the percentage of the surface of the following areas that coincided in the two scans: forehead, left and right cheek, tip of the nose and chin. The software was used to superimpose the scans, automatically determining the best-fit alignment and to calculate the percentage of overlapping surfaces within the following tolerance bands: 0.5 mm to 0 mm and 0 mm to − 0.5 mm, considered highly reproducible; 1 mm to 0.5 mm and − 0.5 mm to − 1 mm, considered moderately reproducible; 1.5 mm to 1 mm and − 1 mm to − 1.5 mm, considered poorly reproducible; and, finally, > 1.5 mm and < 1.5 mm, considered not reproducible. The superimposition of the 3D scans from the three facial scanners was performed pairwise, making a total of three overlays, namely Face Hunter–Vectra, Bellus 3D–Vectra and Bellus 3D–Face Hunter.

### Statistical analysis

As for the statistical analysis of the linear distances (Tr–Na^′^, Na^′^–Prn, Prn–Pog′, and left–right Zyg), four repeated measures ANOVAs were conducted for each of the distances, with the aim of testing whether there were differences between the actual measurements and those obtained via the three scanners, and between the measurements produced by the scanners themselves. Subsequently, Bonferroni's post-hoc comparison was performed for significant differences. The repeatability of the linear measurements was verified by four paired sample t tests.

To test the significance of the percentages of the 3D surfaces of the five different areas analysed, according to the five different tolerance bands (from 0.5 to 0 mm and from 0 to − 0.5 mm, from 1 to 0.5 mm and from − 0.5 to − 1 mm, from 1.5 to 1 mm and from − 1 to − 1.5 mm, and > 1.5 mm and < 1.5 mm), 25 repeated measures ANOVAs were performed. Again, Bonferroni’s post-hoc comparisons were performed in the event of significant differences.

For all tests, the significance level considered was p < 0.05. Analyses were conducted using IBM SPSS v28 software.

## Results

Table 2 reports the linear measurements made on the scans produced by the three different scanners, alongside the anthropometric measurements made directly on the 30 subjects’ faces. The repeatability of the linear measurements is reported in Table 3.

**Table 2:** Linear measurements (mm) obtained via the three different scanners (Face Hunter, Bellus 3D, and Vectra) and direct anthropometric measurements

Subjects	face hunter (MM)				Bellus 3D (MM)				Vectra (MM)				Direct anthropometry (MM)			
	Tr–Na’	Na′-Prn	Prn-Pog′	Zyg-Zyg	Tr–Na′	Na′-Prn	Prn-Pog′	Zyg-Zyg	Tr–Na′	Na′-Prn	Prn-Pog′	Zyg-Zyg	Tr–Na′	Na′-Prn	Prn-Pog′	Zyg-Zyg
1	43.41	44.7	68.14	97.54	43.94	44.78	68.46	97.62	43.28	44.53	68.03	98.35	43.88	44.6	68.16	98.14
2	45.54	41.98	59.01	103.37	45.99	42.43	60.01	102.26	46.46	41.62	58.88	103.45	46.54	41.86	59.36	103.32
3	48.15	58.61	69.94	95.98	49.07	59.77	69.5	95.74	49.19	60.08	68.48	95.64	48.94	59.02	69.62	95.36
4	47.8	59.98	73.31	97.41	47.65	59.97	73.83	98.32	48.16	60.54	73.67	99.67	47.9	59.8	73.18	98.74
5	41.36	51.07	61.62	94.42	40.98	50.62	60.96	95.15	41.19	51.79	61.3	96.86	40.48	51.2	61.98	95.58
6	51.34	55.04	70.86	105.08	53.66	54.19	71.14	106.31	52.8	54.97	70.96	105.14	52.6	54.76	70.28	105.44
7	45.04	56.08	64.81	111.35	46.7	56.34	64.03	112.68	45.29	57.33	64.15	113	44.68	56.64	62.76	112.92
8	54.44	59.67	81.4	100.71	56.41	59.78	80.3	101.38	55.29	59.83	81.09	101.22	55.9	60.2	81.9	101.26
9	41.06	57.8	60.53	102.39	42.43	58	60.25	102.76	42.5	58.32	61.41	102.36	42.06	57.8	60	102.8
10	49.55	61.19	67.76	105.26	51.08	61.41	67.13	105.87	50.08	61.88	67.39	105.51	50.56	61.72	67.02	105.56
11	51.32	52.36	67.89	108.11	51.86	52.53	67.08	108.67	51.88	52.65	67.22	107.52	51.48	52.38	67.12	108.52
12	51.6	55.5	70.84	101.77	51.18	55.5	71.07	102.6	53.22	55.85	70.83	104.34	52.74	55.98	71.745	102.38
13	52.89	51.24	73.98	101.8	52.55	50.35	72.92	101.64	53.38	50.82	73.66	102.46	53.78	51.52	73.78	100.9
14	55.6	51.51	70.01	102.95	56.64	51.64	69.33	101.77	66.68	52.36	69.86	102.93	56.26	51.96	70.03	102.52
15	37.57	49.63	67.93	117.63	38.62	49.38	66.66	118.53	37.7	50.01	67.64	118.41	38	49.68	67.46	118.94
16	31.43	53.63	61.81	105.45	31.64	53.24	61.91	106.52	32.12	53.78	61.36	106.66	31.08	53.96	61.42	105.54
17	48.83	61.75	73.06	104.24	49.94	61.36	71.9	105.66	49.3	62.11	72.16	105.26	48.58	61.56	71.8	105.14
18	39.5	51.26	59.57	94.82	39.69	51.32	59.22	94.15	39.82	51.8	58.92	94.71	39.68	51	59.58	94.86
19	43.02	45.76	66.67	95.56	43.83	46.01	66.13	96.11	43.02	45.91	66.52	95.86	43.1	45.98	66.96	95.04
20	51.4	60.6	63.17	108.86	52.42	59.6	63.38	108.3	53.21	59.75	63.46	109.08	52.02	60.36	62.8	108.6
21	51.29	50.73	72.89	112.01	53.37	50.9	72.6	112.47	51.54	50.9	72.77	113.55	51.62	51.02	72.72	113.74
22	44.53	50.71	65.5	101.19	44.9	50.37	64.79	102.14	45.17	51.69	65.62	101.72	44.86	50.2	65.92	101.36
23	36.83	45.17	68.01	99.01	37.68	44.16	67.41	99.29	37.86	45.04	68.81	100.4	37.2	44.92	68.52	100.52
24	43.77	51.88	76.03	103.87	43.61	52.02	75.98	103.55	43.64	52.81	76.97	105.6	43.72	52.1	76.08	104.52
25	45.12	55.5	70.71	95.33	45.15	55.14	70.16	96.62	44.96	56.33	70.45	96.81	45.02	56.1	69.54	96.88
26	45.6	50.39	68.47	101.1	45.65	49.99	67.88	100.86	45.94	50.9	68.33	101.31	45.94	46.86	68.84	101.14
27	51.54	52.76	63.24	102.79	52.94	52.69	65.68	102.43	51.91	52.97	63.85	102.54	52.54	51.56	62.16	102.1
28	39.28	55.42	66.06	93.78	39.67	56.66	65.05	94.8	39.65	56.95	65.24	94.02	39.26	55.9	66.52	94.26
29	54.07	63.18	72.64	98.87	55.2	63.71	72.06	99.52	54.95	63.78	72.02	99.42	55.3	63.18	71.76	100
30	46.44	52.15	67.72	98.61	47.56	52.25	66.86	99.35	47.53	52.1	67.2	99.96	46.52	51.96	66.96	99.98

**Table 3:** Paired sample *t* test to verify the repeatability of linear measurements

Distance	Significance
Tr–Na′	*p* = 0.603
Na′–Prn	*p* = 0.567
Prn–Pog	*p* = 0.685
Zyg–Zyg	*p* = 0.204

The repeated measures ANOVA test was applied to linear distances, in which the four distances made via the scanners (Face Hunter, Bellus 3D, and Vectra) and the direct anthropometry measurements were compared (Table 4). The test revealed statically significant values for all distances investigated except for the Prn–Pog′ distance (*p* = 0.092), which showed no significant differences among measurement methods. Following the results obtained from the repeated measures ANOVA test, Bonferroni's post-hoc analysis was performed to investigate which of the other comparisons was statistically significant for each linear distance. (Table 5) When comparing facial scans with real measurements, all comparisons were found not to be statistically significant, except for the anthropometry–Face Hunter, in which statistically significant difference was found for the distances Tr–Na′ and Zyg–Zyg. The Tr–Na′ (*p* < 0.001) and Zyg–Zyg distances (*p* = 0.046) were also significantly different when comparing the Face Hunter scanner and the Bellus 3D. The significant differences revealed by comparison of the Face Hunter and Vectra scans were the distances Na’–Prn (*p* = 0.003) and Zyg–Zyg (*p* < 0.001). Finally, comparison between the Bellus 3D scanner and Vectra indicated a statistically significant difference for the distance Na′–Prn (*p* < 0.001).

**Table 4:** Repeated measures ANOVA test on linear measurements

	*p* value
Tr–Na′	*p* = 0.022*
Na′–Prn	*p* = 0.004*
Prn–Pog′	*p* = 0.092
Zyg–Zyg	*p* < 0.001*

*p < 0.05

**Table 5:** Bonferroni′s post-hoc analysis of ANOVA′s statistically significant linear measurements

	Tr–Na′	Na′–Prn	Zyg–Zyg
Direct anthropometry–Face Hunter	*p* = 0.003*	*p* > 0.999	*p* > 0.008*
Direct anthropometry–Bellus 3D	*p* > 0.093	*p* > 0.999	*p* > 0.999
Direct anthropometry–Vectra	*p* > 0.775	*p* > 0.060	*p* > 0.220
Face Hunter–Bellus 3D	*p* < 0.001*	*p* > 0.999	*p* = 0.046*
Face Hunter–Vectra	*p* = 0.071	*p* = 0.003*	p < 0.001*
Bellus 3D–Vectra	*p* > 0.999	*p* < 0.001*	*p* = 0.123

*p < *0.05*

The mean percentage overlap of the 3D-scanned surfaces within the five bands of tolerance for the five different areas is reported in Table 6. The three facial scans were superimposed pairwise, making a total of three overlays, specifically: Face Hunter–Vectra, Bellus 3D–Vectra and Bellus 3D–Face Hunter. As shown in Table 6, almost the 100 per cent of the overlapping areas fell within the tolerance limits for all three comparisons analysed. The area with the fewest values outside the tolerance limits (> 1.5 mm and < − 1.5 mm) the tip of the nose. Although more subjects had a percentage of overlap in the non-reproducible bands for the right and left cheek and forehead, these values never involved more than 5% of the area, except in one comparison, which presented over 5 per cent of overlapping area < − 1.5 mm in the forehead area in the Bellus 3D–Vectra comparison.

**Table 6:** Average percentages of overlapping surfaces of the forehead, left cheek, right cheek tip of nose, and chin areas in the five tolerance bands when comparing Vectra–Face Hunter, Vectra–Bellus 3D and Bellus 3D–Face Hunter scanners

Tolerance ranges	Area	Mean (%)		
		Vectra–Face Hunter	Vectra–Bellus 3D	Bellus 3D–Face Hunter
0,5→0; 0→− 0,5 (mm)	Forehead	57.14	40.31	65.92
1,0→0,5; − 05→− 1 (mm)	Forehead	34.31	34.86	30.41
1,5→1,0; − 1,0→− 1,5 (mm)	Forehead	7.27	14.23	2.4
> 1,5 (mm)	Forehead	0.03	2.15	1.35
< − 1,5 (mm)	Forehead	0.73	5.23	0.02
0,5→0; 0→− 0,5 (mm)	Left cheek	58.58	69.67	72.9
1,0→0,5; − 05→− 1 (mm)	Left cheek	33.93	23.46	22.06
1,5→1,0; − 1,0→− 1,5 (mm)	Left cheek	5.25	5.41	3.8
> 1,5 (mm)	Left cheek	0	0.01	1.1
< − 1,5 (mm)	Left cheek	1.31	1.46	0.19
0,5→0; 0→− 0,5 (mm)	Right cheek	64	67.71	74.03
1,0→0,5; − 05→− 1 (mm)	Right cheek	31.47	25.86	19.41
1,5→1,0; − 1,0→− 1,5 (mm)	Right cheek	4.28	4.87	4.4
> 1,5 (mm)	Right cheek	0	0.04	1.31
< − 1,5 (mm)	Right cheek	0.27	1.47	1.16
0,5→0; 0→− 0,5 (mm)	Tip of the nose	52.06	54.5	86.81
1,0→0,5; − 05→− 1 (mm)	Tip of the nose	42.98	36.5	15.41
1,5→1,0; − 1,0→− 1,5 (mm)	Tip of the nose	4.31	8.63	0.39
> 1,5 (mm)	Tip of the nose	0.02	0.29	0
< − 1,5 (mm)	Tip of the nose	0.08	0.09	0
0,5→0; 0→− 0,5 (mm)	Chin	59.4	59.15	70.13
1,0→0,5; − 05→− 1 (mm)	Chin	30.17	30.76	25.13
1,5→1,0; − 1,0→− 1,5 (mm)	Chin	13.26	6.37	4.87
> 1,5 (mm)	Chin	1.3	3.33	0.05
< − 1,5 (mm)	Chin	0.35	0.28	0.01

Results of repeated measures ANOVA testing of the percentage overlap of the 3D-scanned surfaces within the five ranges of tolerance are reported in Table 7. The chin proved to be the only area in which there were no statistically significant differences between the percentage overlap of 3D-scanned surfaces. There were statistically significant differences in pairwise comparisons between the forehead, the right and left cheek and the tip of the nose within the different tolerance bands, specifically: the forehead and the chin in the bands 0.5 mm to 0 mm and 0 mm to − 0.5 mm (*p* = 0.022 and *p* < 0.001, respectively) and in the bands 1.5 mm to 1 mm and − 1 mm to − 1.5 mm (*p* = 0.008 and *p* = 0.048, respectively); and the left and right cheeks in the bands 1 mm to 0.5 mm and − 0.5 mm to − 1 mm (p < 0.001 and p = 0.001, respectively).

**Table 7:** Repeated measures ANOVA test of the percentage (%) overlap of 3D-scanned surfaces within the five tolerance bands

	Forehead	Left cheek	Right cheek	Tip of the nose	Chin
0,5→0; 0→− 0,5 (mm)	*p* = 0.022*	*p* = 0.007*	*p* = 0.064	*p* < 0.001*	*p* = 0.146
1,0→0,5; − 05→− 1 (mm)	*p* = 0.114	*p* < 0.001*	*p* = 0.001*	*p* = 0.332	*p* = 0.395
1,5→1,0; − 1,0→− 1,5 (mm)	*p* = 0.008*	*p* = 0.555	*p* = 0.911	*p* = 0.048*	*p* = 0.158
> 1,5 (mm)	*p* = 0.405	*p* = 0.276	*p* = 0.188	*p* = 0.341	*p* = 0.418
< − 1,5 (mm)	*p* = 0.046*	*p* = 0.245	*p* = 0.368	*p* = 0.565	*p* = 0.561

*p < *0.05*

Bonferroni post-hoc analysis was performed to verify which of the comparisons were statistically significant (Table 8). All areas, except for the chin, for which no statistically significant differences were found between the overlays of the 3D facial scans, were associated with statistically different values within the bands of 1.5 mm to 1 mm and − 1 mm to − 1 mm. However, the only area to present statistically different values within the tolerance bands considered non-reproducible was the forehead area, where the Vectra–Bellus 3D and Bellus 3D–Face Hunter comparisons produced a p value of 0.044. However, it should be considered that, despite the statistical significance of this value, it is not a large deviation from the threshold of *p* = 0.05.

**Table 8:** Bonferroni post-hoc analysis of the overlap of 3D-scanned surfaces

Tolerance range	Scanner comparison	Areas			
		Forehead	Left cheek	Right cheek	Tip of the nose
0,5→0; 0→− 0,5 (mm)	Face Hunter–Vectra/Vectra–Bellus 3D	*p* = 0.055	*p* = 0.017*	–	*p* = 0.684
	Face Hunter–Vectra/Face Hunter–Bellus 3D	*p* = 0.300	*p* = 0.010*	–	*p* < 0.001*
	Vectra–Bellus 3D/Bellus 3D–Face Hunter	*p* = 0.009*	*p* = 0.437	–	*p* < 0.001*
1,0→0,5; − 05→− 1 (mm)	Face Hunter–Vectra/Vectra–Bellus 3D	–	*p* = 0.011*	*p* = 0.039*	–
	Face Hunter–Vectra/Face Hunter–Bellus 3D	–	*p* < 0.001*	*p* < 0.001*	–
	Vectra–Bellus 3D/Bellus 3D–Face Hunter	–	*p* < 0.011*	p = 0.071	–
1,5→1,0; − 1,0→− 1,5 (mm)	Face Hunter–Vectra/Vectra–Bellus 3D	*p* = 0.078	–	–	*p* = 0.269
	Face Hunter–Vectra/Face Hunter–Bellus 3D	*p* = 0.104	–	–	*p* = 0.168
	Vectra–Bellus 3D/Bellus 3D–Face Hunter	*p* = 0.008*	–	–	*p* = 0.012*
> 1,5 (mm)	Face Hunter–Vectra/Vectra–Bellus 3D	–	–	–	–
	Face Hunter–Vectra/Face Hunter–Bellus 3D	–	–	–	–
	Vectra–Bellus 3D/Bellus 3D–Face Hunter	–	–	–	–
< − 1,5 (mm)	Face Hunter–Vectra/Vectra–Bellus 3D	*p* = 0.052	–	–	–
	Face Hunter–Vectra/Face Hunter–Bellus 3D	*p* = 0.180	–	–	–
	Vectra–Bellus 3D/Bellus 3D–Face Hunter	*p* = 0.044*	–	–	–

Bold indicates *p* < 0.05

## Discussion

This study, designed to compare three different face scanning systems—stereophotogrammetry (Vectra scanner), structured light (Face Hunter scanner), and the TrueDepth camera system available on Apple devices (Bellus 3D application)—found significant differences for all linear averages considered. The only exception was the distance Prn– Pog′, which was statistically similar for all the three systems. Post-hoc analysis of the other distances revealed that the only statistically significant differences to the direct anthropometric measurements were those taken with the Face Hunter scanner, particularly the Tr–Na′ and Zyg–Zyg distance. Comparison of linear measurements on 3D facial scans with direct anthropometry (DA) measurements has proved they are reliable and repeatable, whereas the same measurements on a 2D photograph could lead to errors and inaccuracies due to the lack of the third dimension [11]. These results are similar to those reported by Pellitteri et al. [3], who also found good Prn– Pog′ accuracy but poor Tr–Na′ accuracy, which they ascribed to the location of the Trichion point, near the hairline; indeed, this could make that area difficult for scanners to capture, leading to a poorly defined image and probable measurement errors. Likewise, Aung et al. [18] also reported that all measurements made that included the Trichion point were less reproducible.

In our study, a possible explanation for the Face Hunter facial scanner rendering being the only one with statistically different averages to those obtained directly on the patient’s face could be due to the length of the scanning process. In fact, the Face Hunter needs five static photographs of the patient’s face in five different positions. It therefore requires a longer acquisition time than the Vectra scanner or Bellus 3D application, thereby increasing the probability of involuntary movements. This highlights the importance of studying the accuracy of face scanners in vivo, rather than on mannequins, as minimal variations in facial expression can significantly affect the accuracy of the measurements, reducing reproducibility [1, 3, 7, 11].

The poor reproducibility of the forehead area was also confirmed by the results of our 3D area overlay analysis. Specifically, the forehead showed statistically significant differences in three different tolerance bands: 0.5– > 0 and 0– > − 0.5 (mm), 1.5– > 1.0 and − 1.0– > − 1.5 (mm) and < − 1.5 (mm), considered highly reproducible, poorly reproducible; and not reproducible, respectively. Furthermore, when specifically analysing which overlay combinations produced statistically different values, this was invariably the comparison between Vectra–Bellus 3D/Bellus 3D–Face Hunter. However, it must be considered that more than 90% of the averages of overlapping surfaces between two scans fell within the tolerance band (1.5 mm to − 1.5 mm). In contrast, in the study by Pellitteri et al. [3], less than 80% of the overlap of this area fell within the tolerance margins.

Possible reasons for forehead registration failure were that it is difficult to achieve an absolutely neutral expression, and the proportion of forehead was relatively small for some people [12]. Moreover, for facial scanners, acquiring areas with large curvatures is less reproducible with respect to well defined edges. Indeed, it has been found that estimates of error of 3D images tend to be higher in variables of greater size [3, 11]. Strategies to improve scanning accuracy would therefore involve training the subjects to achieve neutral expression, and checking the subjects’ expression before beginning the scan, as well as checking factors that affect the imaging quality, such as ambient light, and expanding the registration area as much as possible [12].

According to ANOVA, the chin was the area that did not produce differences in the comparison among the three different scanners. That the chin is an easy acquisition area for facial scanners had already been indicated by analysis of the linear distances, in which the Prn–Pog′ was found to be the most accurately reproduced, followed by the right cheek, the tip of the nose, and the left cheek. In fact, analysis of the percentages of overlapping areas showed that, on average, about 60% of the area analysed fell within the tolerance range considered highly reproducible (59.4% in the Vectra–Face Hunter comparison, 59.15% in the Vectra–Bellus 3D comparison, and 70.13% in the Bellus 3D–Face Hunter comparison). Similarly good values in the same tolerance range were also obtained for the tip of the nose and cheeks.

These results are in line with those obtained by Pellitteri et al. [3] However, the percentages of areas within the tolerance range obtained in their study appear to be lower than those in ours. Specifically, the area that achieved the highest percentage was the right cheek, at 59%, followed by the left cheek, at 58%. The chin, despite obtaining relatively low values in the bands considered non-reproducible (3.27% in the > 1.5 mm band and 2.44% in the < − 1.5 mm band), did not exceed 50% in the highly reproducible range. [3]

In contrast to the above results are those reported by Kau et al. [19], who, when two scans were superimposed, found the largest errors in the lower third of the face. According to Kau [19], this occurs because the mandible is a mobile bone, and is therefore subject to muscle contractions that can change its position. The conclusions of the study by Akan et al. [5] are in line with those of Kau et al. [19], namely that points located on curved and small areas such as the eyes and nose are more prone to 3D imaging errors.

All this considered, this study shows how all three scanning systems used (stereophotogrammetry, structured light and smartphone) can be considered accurate means of obtaining 3D facial models. However, the literature agrees that the acquisition modalities of these scanners and, in particular, the scanning speed, is of crucial importance to avoid the introduction of possible errors that may reduce the accuracy of the scans [3–7]. A longer acquisition time, or the need for many movements of the patient’s head during scanning, may increase the possibility of changes in the patient’s facial expression. For these reasons, further studies are needed to investigate whether the acquisition time of a scanner may affect the accuracy of facial scans provided by different scanning systems.

## Conclusions

The conclusions of this study comparing the accuracy of three face scanners with different acquisition systems (stereophotogrammetry, structured light and smartphone) are as follows:

1. All three acquisition systems proved to be effective in capturing 3D images of the face.
2. The Face Hunter scanner is the only scanner that produced statistically significant differences in linear measurements for the distances Tr–Na’ and Zyg–Zyg with respect to direct anthropometric measurements, although all scanners accurately reproduced the Prn- Pog′ distance.
3. Areas overlap analysis between scanners confirmed the accuracy of all systems, with more than 90 per cent of each area analysed falling within the highly reproducible band.
4. The chin was the most accurately reproduced, with no differences among scanners, while the forehead proved to be the least accurately reproduced by all scanners.

## Data Availability

The datasets used and/or analysed during the current study are available from the corresponding author on reasonable request.
